# Corticosteroid treatment prediction using chest X-ray and clinical data

**DOI:** 10.1016/j.csbj.2023.11.057

**Published:** 2023-12-07

**Authors:** Anzhelika Mezina, Samuel Genzor, Radim Burget, Vojtech Myska, Jan Mizera, Aleksandr Ometov

**Affiliations:** a Brno University of Technology, FEEC, Dept. of Telecommunications, Technicka 12, Brno, 616 00, Czech Republic; b Center for Digital Health, Palacky Univesity Olomouc, Faculty of Medicine and Dentistry, Hnevotinska 976/3, Olomouc 779 00, Czech Republic; c Electrical Engineering Unit, Faculty of Information Technology and Communication Sciences, Tampere University, Tampere, 33720, Finland

**Keywords:** Image classification, Chest X-ray images, Vision transformer, Treatment prediction, Clinical data, Post-acute COVID-19

## Abstract

**Background and Objective:**

Severe courses of COVID-19 disease can lead to long-term complications. The post-acute phase of COVID-19 refers to the persistent or new symptoms. This problem is becoming more relevant with the increasing number of patients who have contracted COVID-19 and the emergence of new virus variants. In this case, preventive treatment with corticosteroids can be applied. However, not everyone benefits from the treatment, moreover, it can have severe side effects. Currently, no study would analyze who benefits from the treatment.

**Methods:**

This work introduces a novel approach to the recommendation of Corticosteroid (CS) treatment for patients in the post-acute phase. We have used a novel combination of clinical data, including blood tests, spirometry, and X-ray images from 273 patients. These are very challenging to collect, especially from patients in the post-acute phase of COVID-19. To our knowledge, no similar dataset exists in the literature. Moreover, we have proposed a unique methodology that combines machine learning and deep learning models based on Vision Transformer (ViT) and InceptionNet, preprocessing techniques, and pretraining strategies to deal with the specific characteristics of our data.

**Results:**

The experiments have proved that combining clinical data with CXR images achieves 8% higher accuracy than independent analysis of CXR images. The proposed method reached 80.0% accuracy (78.7% balanced accuracy) and a ROC-AUC of 0.89.

**Conclusions:**

The introduced system for CS treatment prediction using our neural network and learning algorithm is unique in this field of research. Here, we have shown the efficiency of using mixed data and proved it on real-world data. The paper also introduces the factors that could be used to predict long-term complications. Additionally, this system was deployed to the hospital environment as a recommendation tool, which admits the clinical application of the proposed methodology.

## Graphical abstract

**Figure:**
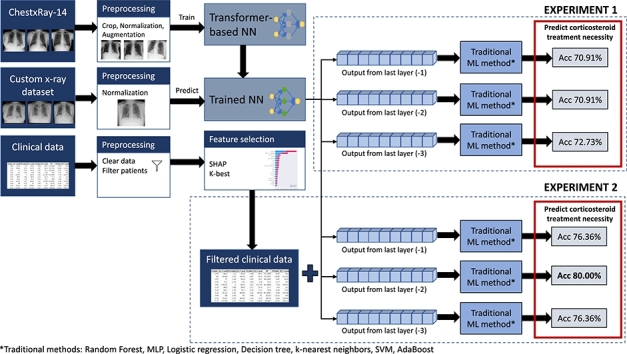

## 1 Introduction

Since 2020, the pandemic SARS-COV-2 (COVID-19) affected the lives of all people in the world. Although some cases have been reported as asymptomatic COVID-19 infections [1], almost 7 million cases (November 2023) of COVID-19 were fatal according to World Health Organization (WHO) statistics.

Despite rapid and continuous improvements in prevention, diagnostics, and treatment, dealing with possible consequences and complications in severe disease courses is still necessary. Those complications (including thromboses, decompensation of comorbid diseases, and finally, pulmonary fibrosis (PF)) may lead to increased morbidity and mortality [2]. One of the common complications of severe COVID-19 courses may be the subsequent development of PF [3]. Such complications can be detected during the post-acute phase of COVID-19 (so-called long COVID). Preventive treatment with corticosteroids (CS) is one of the ways to decrease the risk.

Nonetheless, not all patients benefit from CS treatment, and, moreover, this medication is connected to numerous severe side effects [4], [5], [6], [7]. Some studies found that non-selected applications of CS treatment may be harmful [8], [9], and it is still unclear which patients may receive this therapy.

**Currently, there is still a lack of studies that would make personalized recommendations on which patients will benefit from the CS treatment and who will not.** Additionally, not so many works use the combined clinical data for analysis because of the difficulty of collecting them. According to WHO statistics, there are 100,000 infections on average a week (at least in November 2023), and there is a higher risk of development of PF after the severe acute phase of COVID-19 [10].

The objective of this paper **is to find the relation between a patient's clinical condition in the post-acute phase of COVID-19 at the beginning of treatment and the probability of benefit from CS treatment using clinical information and chest X-ray image of the patient**. Sometimes, patients may not be recommended to receive this medication and can avoid possible side effects.

To achieve this goal, we retrospectively analyzed the data from 273 patients showing the results of (non-)application of CS treatment and applied several techniques to preprocess this clinical data. We further proposed an ML-based and DL-based methodology that predicts whether the CS treatment is recommended or not based on the patient's clinical data and the CXR. The application of artificial intelligence (AI) is an appropriate way because of its capability to process a large number of parameters and to find the relations in these data.

The described experiment has two parts:

1. CXR images have been used to predict whether the CS treatment is necessary. This part is performed using our novel deep learning (DL) architecture;
2. CXR images and data retrieved from spirometry, blood information, and anamnesis of the patient's state of health were processed using our DL architecture and ML with a preprocessing of features.

**Fig. 1:**
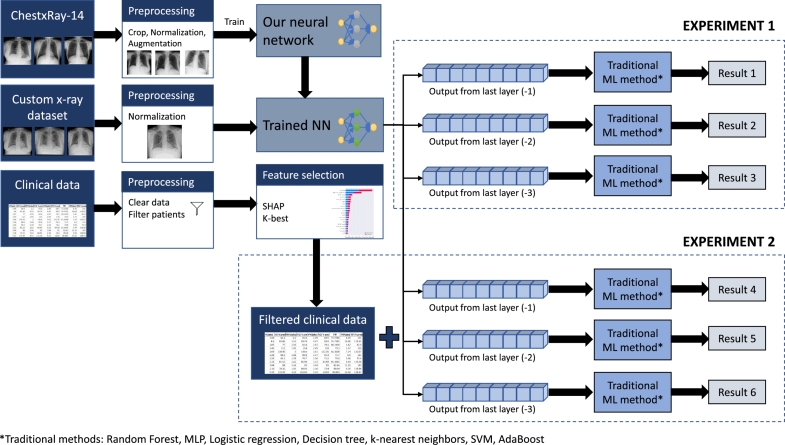
The overall scheme of experiments for proposed recommendation system of CS treatment.

During these experiments, we show that such a combination of information, such as clinical data and CXR images, provides about 8% better accuracy than when it is used separately. The overall scheme of the experiment is depicted in Fig. 1.

The main contributions of this paper are:

• Developed DL-based approach for treatment prediction for one of the most frequent long-term COVID-19 complications. The methodology is unique, and there is no comparable one published for this disease yet.
• The proposed approach combines traditional machine learning methods for clinical data processing and our architecture of the neural network, which consists of Vision Transformer and InceptionNet, for analysis of CXR images.
• This study shows that combining clinical data and CXR images can give more accurate results than when used separately.
• A wide range of parameters from patients' anamnesis was analyzed, and the most statistically significant parameters were identified.
• The proposed framework was integrated into the internal hospital system as a part of the recommendation tool.
• The dataset with clinical data and CXR from patients is introduced and available in [11].

The rest of the paper is structured as follows. Section 2 represents related work and describes relevant solutions based on utilizing mixed data and predicting treatment of other health problems. Next, Section 3 provides more details about the introduced methodology and neural network and the following prediction of CS necessity using clinical and CXR data. Further, Section 4 shows and discusses the experiment results. The last Section 5 concludes the paper.

## 2 Related work

This Section represents the latest works and approaches that focus on applying AI in the related research fields to the stated problem. Firstly, the relevant DL methods for CXR processing are introduced. Secondly, the overview of methods for processing mixed data is presented. Lastly, the application of ML for treatment prediction is provided.

### 2.1 Chest X-ray image analysis

CXR and Computed Tomography (CT) are mandatory and the most important diagnostic methods in pulmonology [12]. Since the amount of radiation received from a CXR is approximately 70× lower than the radiation received from CT [13], the CXR is still preferred for a general patient's examination due to possible consequent risks. Therefore, the widespread task in the research field is the analysis of CXR images, often performed using DL methods. These methods can be useful as an assistant tool for the radiologist [14].

One of the frequently used ways to construct the neural network for that is to use pre-trained models, such as ResNet [15], DenseNet [16], InceptionNet [17], etc., and to add some new blocks or modules, which help to adapt the model for a given problem. For example, the VGG16 with attention module distinguishes between COVID-19, viral, bacterial pneumonia, and normal [18]. Or, the same classes were detected using EfficientNet-B5 with noisy student in [19]. Also, the approach [20] utilizes the pre-trained VGG16 model and several augmentation techniques (conventional method, mixup, and Random Image Cropping and Patching (RICAP)) to prevent the overfitting of the model.

One of the first approaches related to COVID-19 detection is described in [21]. The authors proposed an ensemble deep learning model consisting of Densenet-121, Resnet-50, InceptionNet, Inception-Resnet, XCeption, and EfficientNet-B2. As the input to the model, four images were generated: cropped and uncropped with sizes of 224×224 px and 331×331 px. The work results demonstrate that the achieved accuracy of 83% is comparable with the consensus of experienced thoracic radiologists.

The strategy of several branches is also applied in another work [22]. The authors used 20 individually trained deep neural networks to detect COVID-19 and non-COVID-19 pneumonia. This ensemble model achieved an AUC of 0.92. The mentioned models can also be modified, as was done in [23]. The MobileNet network was adapted here for CXR image classification to avoid the gradient vanishing problem and overfitting. Another work [24] performed experiments for COVID-19 detection using pre-trained models EfficientNetB1, NasNetMobile, and MobileNetV2 and achieved an accuracy of 96%. EfficientNet was also applied for COVID-19 detection in [25]. The proposed method has incorporated uncertainty into EfficientNetB3.

Some works describe the models, combining some pre-trained well-known models, as done in [26]. The authors proposed a model consisting of MobileNetV2 and VGG16, and the final classification is performed with the concatenation of these two branches. The authors classified CXR images into COVID-19, Normal, Pneumonia Viral, and Pneumonia Bacterial. InceptionNetV3 can also be applied for this task [27]. Additionally to that, CycleGAN was used for data augmentation. Also, a possible way is to use the convolutional network, which consists of convolutional and max pooling layers. According to the authors of work [28], it is possible to achieve an accuracy of 99% for COVID-19 detection.

According to the studied literature, many approaches focus on processing CXR obtained from the acute phase of COVID-19 and detecting viral and bacterial pneumonia and COVID-19. However, there is no attention to the post-acute phase of this disease.

### 2.2 Analysis of mixed medical data

In medical research, data analysis can be done over images, clinical data, or a combination of both. Such a combination of data can give much more information, and consequently, it can increase the accuracy of AI methods [29]. Such application is also actual in a pandemic, where predicting the possible complications is necessary. It can assist in prescribing treatment or clinical outcomes and help organize hospital resources.

With this motivation, the authors of work [30] proposed a methodology that uses mixed data to identify patients at risk of severe consequences, which can lead to intensive care or death. The article described three data-processing approaches: handcrafted, hybrid, and end-to-end learning. The best-achieved accuracy is 76.9%, which is produced by a hybrid method.

Another work [31] uses mixed data in an end-to-end manner. The proposed neural network consists of two branches: CNN, which processes CXR images, and MLP, which consists of two dense layers for numerical data processing. The goal is to differentiate between COVID-19 and non-COVID-19 patients. According to the results, the model has achieved 95.4% accuracy.

The comparison of three different scenarios, diagnosis of COVID-19 using clinical data with a fully connected neural network, CXR images with EfficientNetB7, and a combination of both, is introduced in [32]. The authors used data from 270 patients. The most accurate case is the combination of clinical data and CXR images, which achieved an accuracy of 97%.

The disease severity and progression are also described in [33]. For the experiment, the EfficientNet for image-based severity prediction and a neural network with some dense layers for clinical-data-based severity prediction are used to perform the final severity prediction. Combined progression prediction is performed using a so-called survival forest for output of the dense layer with 256 neurons from the previously mentioned models for images, clinical parameters, and chest CXR severity scores. The results show that the image and clinical data combination performs better for severity prediction (ROC-AUC 0.792) and progression prediction (C-index 0.752) on the external test set.

Another application of DL to mixed data is represented in [34]. The methodology consists of the following steps: 1) the 3D CNN extracts the features from CT images, the clinical data are concatenated, 2) dimensionality reduction is performed with the Principal Component Analysis (PCA) method, 3) final classification is performed using CatBoost. The algorithm is aimed to predict categories of patients: who needed intensive care unit admission or were dead and who were healed and moved to non-COVID wards for further care. The achieved results are promising: AUC of 0.949.

Currently, most studies using mixed data focus exclusively on the acute phase of COVID-19 treatment. However, no approach analyzes data from the post-acute phase of COVID-19. In this way, our work fills the gap in research by providing analysis using AI of patients' CXR and clinical data from the post-acute phase.

### 2.3 Treatment recommendation

Another research question is whether it is possible to predict the response to some treatment or an optimal dose of medication using AI.

One of our latest research works [35] is primarily focused on CS treatment prediction, but it is based only on clinical data of patients. This approach compared several ML algorithms, such as Logistic Regression, k-NN, Decision Tree, XGBoost, Random Forest, SVM, MLP, AdaBoost, and LGBM, for the mentioned problem. The best-achieved results belong to the decision tree with a balanced accuracy of 73.52%.

Some works have already been focused on this task, but for other fields of medicine. One of the popular fields of research is related to cancer. For example, the work [36] proposes the two-staged DL framework, which consists of tumor segmentation and response prediction. The results show that the segmentation component is essential to response prediction.

Another approach combines the information from the CT image and clinical data [37]. The article retrospectively analyzes the outcome predictions for individualized radiotherapy doses. The proposed model predicts treatment failures with a C-index of 0.72.

Also, ML was applied to predict insufficient response to methotrexate in patients with Rheumatoid Arthritis [38]. The results show logistic regression is the most successful in this task, and achieved an AUC of 0.77. On the other hand, some works focused on predicting the outcome of treatment for COVID-19 [39]. For example, work [40] proposes a DL framework with Whale Optimization Algorithm (WOA), which utilizes CT images and some additional information about patients (age, infection stage, etc.) and is aimed to predict the patient's response to treatment during the acute phase.

### 2.4 Related work summary

All approaches mentioned in this section are summed up in Table 1. Considering the studied literature and the current state of technologies, it can be concluded that there is no work focusing on retrospective analysis based on clinical data and CXR images of patients' COVID-19 post-acute phase. Additionally, no ML/DL approach would recommend treatment for one of the most severe long-term post-COVID complications, except our work. This way, we aim to fill the gap in the research field because our work represents the analysis of such data and provides a unique dataset with a combination of the clinical data and CXR images and a detailed statistical evaluation of the proposed methodology.

**Table 1:** Comparison of related works.

Ref.	Problem solved	Method	Results
Medical analysis using CXR data (classification)			

[23]	COVID-19, bact.pneumonia, viral pneumonia, tuberculosis, normal	Modified MobileNet	Accuracy 0.997
	COVID-19, Non-COVID-19 infection, normal		Accuracy 0.996
	COVID-19, Non-COVID-19 pneumonia, tuberculosis, normal		Accuracy 0.999

[24]	COVID-19, viral pneumonia, normal, lung opacity	EfficientNetB1	Accuracy 0.961

[25]	COVID-19, normal, pneumonia	EfficientNet-B3+Monte Carlo	Accuracy 0.980
	COVID-19, normal		Accuracy 0.994

[26]	COVID-19, normal, pneumonia	MobileNetV2 + VGG16	Accuracy 0.965
	COVID-19, normal, bacterial pneumonia, viral pneumonia		Accuracy 0.902

[27]	COVID-19, non-COVID-19	Inception-CycleGAN	Accuracy 0.942

[28]	COVID-19, normal, bacterial pneumonia, viral pneumonia	22-layers CNN	Accuracy 0.912
	COVID-19, normal, bacterial pneumonia		Accuracy 0.942
	COVID-19, normal		Accuracy 0.991

[21]	COVID-19, non-COVID-19	Deep learning ensemble model	Accuracy 0.83

[22]	COVID-19 pneumonia, non-COVID-19 pneumonia	Deep learning ensemble model with 20 branches	AUC 0.92

Medical data analysis using mixed data			

[30]	COVID-19 mild or severe	GoogleNet + SVM	Accuracy 0.769

[31]	COVID-19 and non-COVID-19	CNN+MLP	Accuracy 0.963

[32]	COVID-19 and non-COVID-19	EfficientB7+MLP	Accuracy 0.97

[33]	Disease severity (critical/non-critical)	EfficientNetB0+MLP	AUC 0.792
	Progression prediction (time-to-event outcome)	EfficientNetB0+survival forest	C-Index 0.752

[34]	Severity (non-ICU and ICU)	CNN+CatBoost	AUC 0.949

Prediction of treatment			

[35]	Response to CS treatment in post-acute COVID-19	Decision tree	Balanced accuracy 0.735

[36]	Pathologic complete response	Convolutional Encoder-Decoder	AUC 0.97

[37]	Predicting treatment failures	Encoder-decoder + regression model	C-Index 0.72

[38]	Prediction of insufficient response to methotrexate	Logistic regression	AUC 0.77

[40]	Prediction of response to COVID-19 treatment	CNN for CT segmentation + SVM + WOA	Accuracy 0.971

## 3 Methodology

This section introduces a DL-based approach to recommend preventive CS treatment for one of the most severe long-term post-COVID-19 complications – PF.

First, data are preprocessed, including image processing and selection from the clinical data. Then, the recently proposed our neural network and the following comparison with other architectures are described. Finally the two scenarios of experiments: 1) with only CXR images, and 2) a combination of CXR and clinical data, are introduced.

Generally, the experiment consists of the following steps (see Fig. 1):

1. Training our neural network on open large dataset ChestX-ray14 [41] and comparing it with other well-known architectures to select the most successful architecture for further use;
2. Testing trained model selected from the previous step on given CXR images to determine the necessity of CS (experiment 1);
3. Statistical evaluation and selection of most significant parameters from clinical data;
4. Application of DL and ML to combined data: CXR image and selected parameters from clinical data (experiment 2).

The dataset [11] and source code [42] are available online.

### 3.1 Data preparation

One of the main parts of ML methods is data preparation. Generally, this phase consists of CXR image preprocessing, parameter selection, and labeling.

#### 3.1.1 Custom dataset

In this work, the new dataset with CXR images and clinical data of patients from post-acute COVID-19 is introduced and provided by University Hospital Olomouc, Czech Republic. Patients' data were collected during the initial check-up, which is 4 – 12 weeks after the acute onset of COVID-19. All of the examinations, including blood tests, CXR, and pulmonary function tests, were performed at the same time.

The dataset has a total of 273 patients, where the information about the necessity of CS is given: 141 patients will not benefit from CS treatment, and 132 will benefit from it. It is essential to note that all of the patients requiring CS treatment had pneumonia during the acute phase of COVID-19. In addition, all of those patients had persisting pulmonary involvement. However, in a significant proportion of the patients, there was spontaneous regression of the lung damage. The model aimed to identify those with profit from CS therapy. This dataset includes more than 100 parameters, including blood test values, pulmonary function test (spirometry), the patient's anamnesis, clinical condition, and X-ray images as summarized in Table 2.

**Table 2:** Description of patients' clinical data from the custom dataset.

Number of patients	273		
Demographic and habits			

Attributes	Values		

Gender	Male	Female	
	166	107	

Age	STD	Mean	Minimum-Maximum
	11.18	64.38	30-90

Body parameters			

Weight (kg)	15.87	88.03	57-136

Height (cm)	9.84	170.01	145-198

BMI	4.98	30.47	20.75-47.37

Therapy and lung damage			

Attributes	Number of patients		
	Yes	No	

Hospitalized	222	51	

Oxygen (O2)	179	94	

Remdesevir	22	251	

CS			

During hospitalization	99	174	

Post-COVID-19 treatment	92	181	

Another diagnostics	4	269	

HRCT – lung damage			

Interstitial involvement	49	224	

Inflammatory changes	118	155	

Persistent health issues			

Attributes	Number of patients		
	Yes	No	N/A

Dyspnea	188	84	1

Cough	93	179	1

Fatigue	77	195	1

Olfactory loss	39	234	0

Gastrointestinal problems	68	205	0

COVID-19 Testing			

Attributes	Number of patients		
	Positive	Negative	N/A

IgM (qualit.)	219	47	7

IgG (qualit.)	264	2	7

Vaccination			

Attributes	Number of patients		
	Yes	No	N/A

1st dozen	11	210	52

2nd dozen	3	206	64

3rd dozen	1	90	182

Notably, several patients had negative or N/A results for COVID-19 testing – anti-SARS-CoV-2 IgG. COVID-19 was diagnosed with very suspicious clinical symptoms, radiology images, and the presence of anti-SARS-CoV-2 IgM antibodies (only in non-vaccinated individuals at the beginning of the epidemic).

#### 3.1.2 Parameters selection

The parameter selection from the clinical data represented with quantitative and categorical numbers is essential for avoiding overfitting. The clinical data consist of information taken at the time of initiation of post-COVID treatment. In this step, the parameters are pre-selected based on two statistical methods and manual selection. The feature selection is depicted in Fig. 3.

**Fig. 2:**
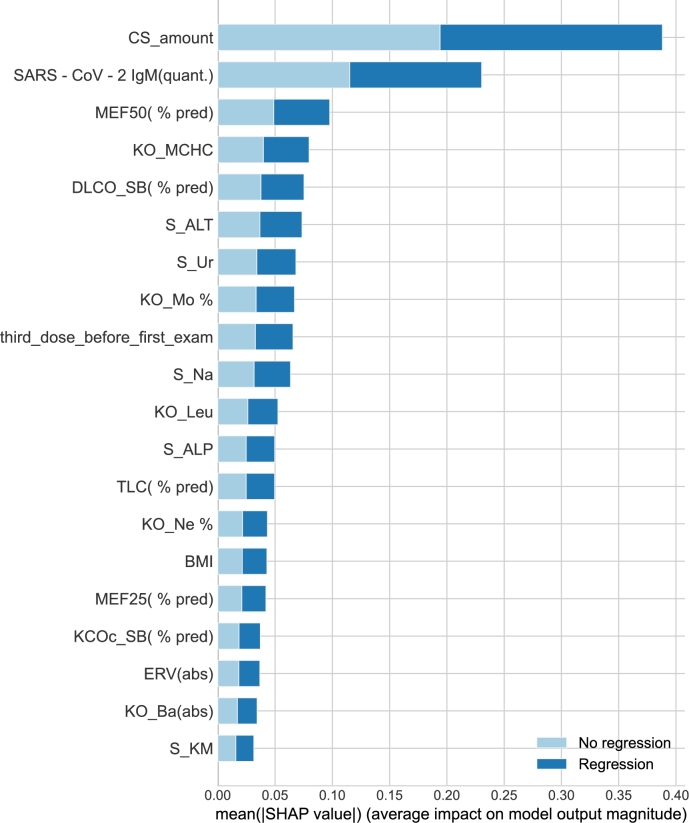
Feature importance analysis based on SHAP method using the decision tree.

The first one is SHapley Additive exPlanations (SHAP) [43], which explains the feature importance for a given ML method, i.e., the Decision Tree in our case. The output is depicted in Fig. 2. As can be seen, the amount of CS received during the treatment (i.e., dose × period) (CS_amount) and Immunoglobulin M antibodies (IgM) (SARS-CoV-2 IgM(quant.)) values from blood tests significantly impact CS treatment. IgM indicates an early immune response after infection in the body.

**Fig. 3:**
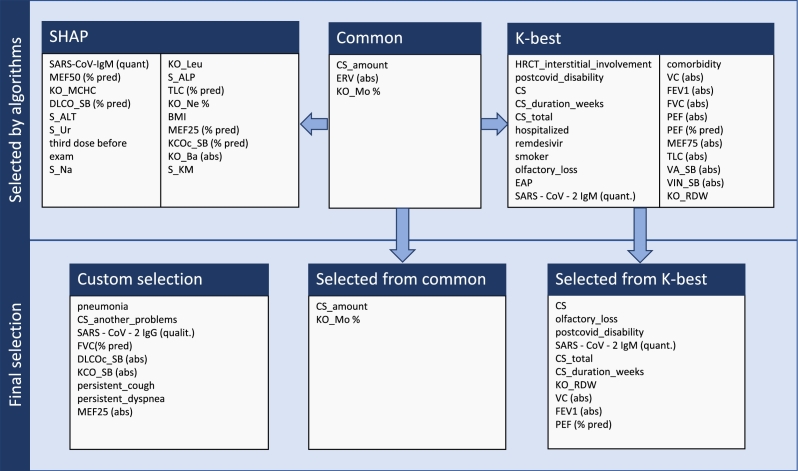
Selection of clinical parameters from custom dataset.

Another statistical method used was *k*-highest scores based on *p*-values [44]. The common parameters of both methods are the amount of used CS (CS_amount), peripheral blood monocytes % (KO_Mo %), and Expiratory Reserve Volume (ERV) (ERV(abs)) values. From the *k*-best method following features were selected: CS use (CS), olfactory loss (olfactory_loss), post-COVID disability (postcovid_disability), level of specific anti-SARS-CoV-2 immunoglobulin M antibodies (SARS-CoV-2 IgM(quant.)), the dose of CS (CS_amount), duration of CS (CS_duration_weeks), red cell distribution width (RDW) (KO_RDW), vital capacity (VC) (absolute) (VC(abs)), forced expiratory volume in 1 second FEV1 (absolute) (FEV1(abs)), and peak expiratory flow (PEF) (% of predicted) (PEF (% pred)).

Additionally, some other parameters were pre-selected manually after series of trials to achieve the best effect of CS prediction. These parameters correlate with the severity of the pneumonia and impact the persisting pulmonary damage, leading to an indication of CS treatment. A brief description of selected ones is introduced below:

• Pneumonia – patients with this variable have had COVID-19 pneumonia;
• CS another problems – patients received CS treatment due to other indications;
• SARS-CoV-2 IgG (qualit.) – presence of IgG antibodies against COVID-19;
• FVC (% pred) – forced vital capacity (% of predicted values);
• DLCOcSB (abs.) – lung diffusing capacity for carbon monoxide (absolute values), which is a transfer factor (i.e. correlate of alveolar volume);
• KCOcSB (abs.) – lung diffusing capacity for carbon monoxide (absolute values), which is a transfer coefficient (i.e. correlate of alveolar diffusion);
• Persistent cough – presence of long-lasting cough after COVID-19 (for more than 4 weeks);
• Persistent dyspnea – presence of long-lasting dyspnea after COVID-19 (for more than 4 weeks);
• MEF25 (abs.) – maximal expiratory flow at 25% of vital capacity.

The final set of features contains the following: Presence of pneumonia at the acute COVID-19 phase, comorbidity, CS use, olfactory loss during the acute COVID-19 phase, post-COVID-19 disability, SARS-CoV-2 IgG(qualit.), SARS-CoV-2 IgM(quant.), amount of used CS, total used CS, duration of CS use in weeks, RDW, VC(abs), FVC(% pred), FEV1(abs), Mo %, PEF(% pred), DLCOc(abs), KCOc(abs), persistent cough, persistent dyspnea and MEF25(abs).

#### 3.1.3 Data labeling

The clinical data also contains information about the objective radiological score, which reflects the lung damage regression in the range of 0 (immutable state) to 10 (complete regression). This parameter gives information on whether the patient's health has improved. Patients with scores between 0 and 6 are not considered to have significantly improved their health status. For the values 7-10, it is considered that their status improved. Considering this information, it was possible to determine the necessity of CS, i.e., whether the patient will benefit from the CS treatment. The dataset was split into training and testing subsets. The training set has 218 patients (111 – not recommended, 107 – recommended). The testing set has 55 patients (30 – not recommended, 25 – recommended).

#### 3.1.4 Chest X-ray image preparation

The CXR images were cropped to the area of interest for all experiments. This step uses a pre-trained U-Net [45] model for lung segmentation, used in [21]. This step helps the neural network to focus on the lungs instead of considering the surrounding area.

Another important step is data augmentation to reduce the model's possible overfitting. For this purpose, transformations (e.g., translation, rotation, and zoom) were applied to extend the dataset during the training.

This work uses two datasets with CXR images: the ChestX-ray14 dataset [41] for pre-training and our custom dataset for CS prediction.

ChestX-ray14 has a total of 112,120 images with 14 pulmonary diseases: Atelectasis, Cardiomegaly, Consolidation, Edema, Effusion, Emphysema, Fibrosis, Hernia, Infiltration, Mass, Nodule, Pleural thickening, Pneumonia, and Pneumothorax. Among the mentioned diseases, some images do not have any disease. It is one of the most frequently used datasets in this field of research. However, the images with at least one pulmonary disease were selected for this experiment. The dataset was divided into training (36,024 images) and testing (15,735 images) parts according to the official dataset split. Also, the validation set is 20% of the training set.

### 3.2 Neural network for feature extraction from X-ray images

A neural network is selected for image processing in this part of the experiment. First, the description of our architecture of the neural network [46] is presented. Then, a comparison with other well-known architectures is performed.

#### 3.2.1 Architecture

The recently proposed neural network is aimed to perform the classification task over CXR images. The first step is to classify images from the ChestX-ray14 dataset, which is prepared for multilabel classification. That is why the following description will be focused on multilabel classification. The overall scheme is introduced in Fig. 5.

**Fig. 4:**
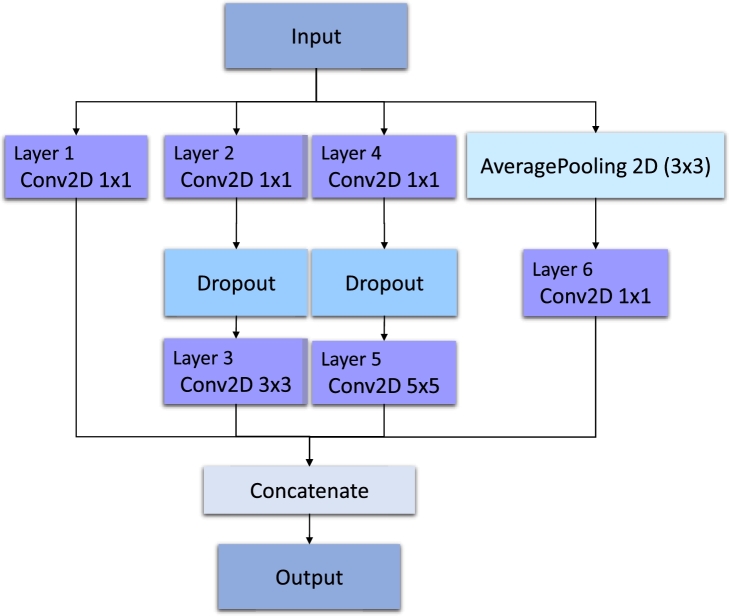
Scheme of the inception module.

This architecture applies the strategy of two branches to capture global and local features. This concept has been widely used in literature [26], [47], [48]. On the one hand, using only one branch is not so effective, according to the results presented in the following sections. On the other hand, applying more branches increases the model's complexity, making it more computationally demanding. That is why the optimal solution in this case is using two branches. The first branch aims to extract the global features and is represented with InceptionNetV3 [17]. After InceptionNetV3, GlobalMaxPooling, Dense, and Dropout layers are used for the final classification. The second branch consists of four parts. First, feature extraction is performed with three inception modules. Fig. 4 shows the architecture of the inception module. After the inception modules, the Vision Transformer (ViT) [49] was utilized for the following processing. Fig. 6 shows the architecture of ViT in more detail.

**Fig. 5:**
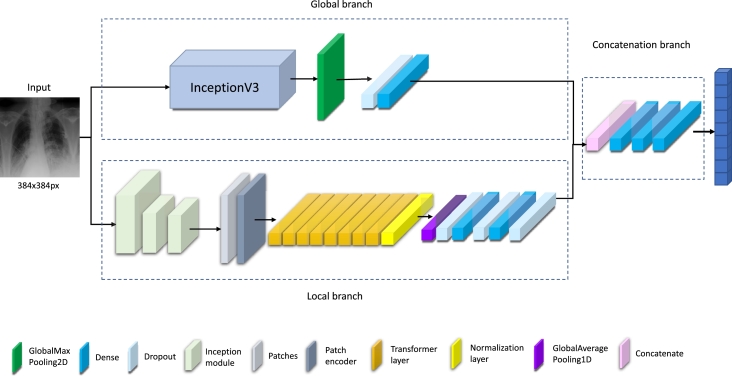
Scheme of the proposed neural network.

**Fig. 6:**
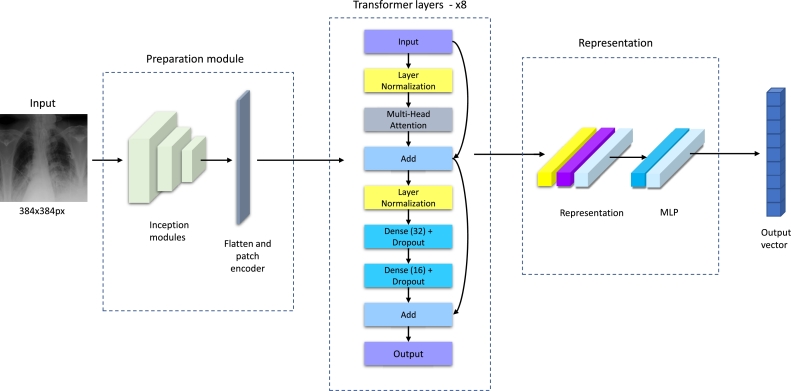
Scheme of the transformer network used in the proposed architecture.

The concatenation of two branches consists of three dense layers with 64, 32, and 14 units to provide the final results. The last layer has a sigmoid activation function since it is necessary to perform the multilabel classification.

Considering that the dataset is slightly imbalanced, the Asymmetric Loss (ASL) function was used [50]. Additionally, it was modified: instead of binary cross-entropy, the focal loss function was applied. Mathematically, it has a unified formula (more details can be found in the original paper) [50]:

(1) $$ASL=\left\{ \begin{array}{c} L_{+}={\left(1-p\right)}^{\gamma_{+}}\log(p),\\ L_{-}={\left(p_{m}\right)}^{\gamma_{-}}\log(1-p_{m}), \end{array}\right.$$

where $L_{+}$ and $L_{-}$ are the positive and negative loss parts, *p* is the output probability from the network, $p_{m}$ is the shifted probability, *γ* is the focusing parameter. The set up parameters are $\gamma_{-}$ is 5, $\gamma_{+}$ is 1, clip is 0.001.

The focal loss function is described in [51]:

(2) $$FL(p_{t})=-\alpha_{t}{\left(1-p_{t}\right)}^{\beta }\log(p_{t}),$$

where $p_{t}$ is the model's estimated probability for the class with label y=1, $\alpha_{t}$ is the balancing factor, and *β* is the modulating factor.

#### 3.2.2 Comparison with traditional methods

To compare the proposed model with other methods, some well-known architectures for image classification were selected: DenseNet121 [16], EfficientNetB4 [52], InceptionNetV3 [17], ResNet101 [15] and VGG16 [53]. These models were also trained on the ChestX-ray14 dataset to detect diseases in CXR. The following values were used for hyper-parameter tuning for all the mentioned architectures, including our architecture: batch size – 8, number of steps per epoch – 1000, number of epochs – 100, optimizer – AdamW, learning rate – 0.0001, weight decay – 0.00001. All mentioned methods used as a baseline apply the binary cross-entropy as a loss function.

Table 3, Table 4 represent the results of trained models. The perfect and maximum value for each metric is 1. Based on the results, it can be concluded that the proposed model achieves better accuracy than other neural network architectures with AUC – 0.7952, specificity – 0.7326, accuracy – 0.7334, precision – 0.2541, and F1 score – 0.3478. The EfficientNetB4 model has worse results than the proposed model but has achieved better results among other methods from the baseline. It has an AUC of 0.7772, sensitivity of 0.7079, specificity of 0.7221, accuracy of 0.7223, precision of 0.2419, and F1 score of 0.3324. On the other hand, the worst results were achieved by ResNet101 for such metrics as accuracy (0.7520) and sensitivity (0.7011). Table 3, Table 4 also show the results for InceptionNetV3, used in the proposed model. It performed with an AUC of 0.7522, sensitivity of 0.7073, specificity of 0.6869, accuracy of 0.6864, precision of 0.2314, and F1 score of 0.3171. As can be seen, the application of InceptionNetV3 independently has worse results for all metrics than the proposed model. From this fact, it can be concluded that the application of ViT and asymmetric loss function is effective for this field of research.

**Table 3:** Evaluation of the achieved results by all methods.

Method	AUC	Sensitivity	Specificity	Accuracy	Precision	F1 Score
DenseNet121	0.7667	0.7169	0.6965	0.7022	0.2324	0.3234
EfficientNetB4	0.7772	0.7079	0.7221	0.7223	0.2419	0.3324
ResNet101	0.7520	0.7011	0.6917	0.6975	0.2320	0.3232
VGG16	0.7797	**0.7427**	0.6930	0.6978	0.2433	0.3357
InceptionNetV3	0.7522	0.7073	0.6869	0.6864	0.2314	0.3171
Our model	**0.7952**	0.7281	**0.7326**	**0.7334**	**0.2541**	**0.3478**

**Table 4:** Detailed AUC results achieved with all methods for each disease.

Pathology	DenseNet121	EfficientNetB4	ResNet101	VGG16	InceptionNetV3	Our model
Atelectasis	0.7325	0.7421	0.7450	0.7587	0.7379	**0.7610**
Cardiomegaly	0.8513	0.8594	0.8518	0.8684	0.8423	**0.8789**
Consolidation	0.6635	0.6663	0.6665	0.6890	0.6614	**0.6983**
Edema	0.8208	0.8187	0.8128	0.8253	0.7902	**0.8313**
Effusion	0.7886	0.7858	0.7944	0.8058	0.7815	**0.8062**
Emphysema	0.8313	0.8490	0.8088	0.8673	0.8147	**0.8865**
Fibrosis	0.7921	0.8263	0.7983	0.7888	0.7999	**0.8330**
Hernia	0.8549	**0.8910**	0.6222	0.7860	0.7138	0.8787
Infiltration	0.6791	0.6850	0.6762	**0.7035**	0.6727	0.6978
Mass	0.7797	0.7803	0.7865	0.8073	0.7773	**0.8123**
Nodule	0.7306	0.7442	0.7427	0.7541	0.7216	**0.7657**
Pleural Thickening	0.7011	0.7222	0.7181	0.7258	0.7138	**0.7376**
Pneumonia	0.6632	0.6644	0.6594	**0.6786**	0.6684	0.6778
Pneumothorax	0.8446	0.8461	0.8455	0.8576	0.8346	**0.8676**

Considering the above-mentioned results, our model is the most suitable for the following experiments.

### 3.3 Experiments with custom dataset

This section describes the experiments on our custom dataset containing CXR images and clinical data. As mentioned in previous sections, the experiment's goal is to determine whether treating the patient with CS is recommended. This experiment is divided into two parts. The first one is aimed at experimenting only on CXR images. The second part is designed to apply CXR images and clinical data. The pre-trained model from section 3.2 is used for both parts.

#### 3.3.1 Classification of chest X-ray images

**Fig. 7:**
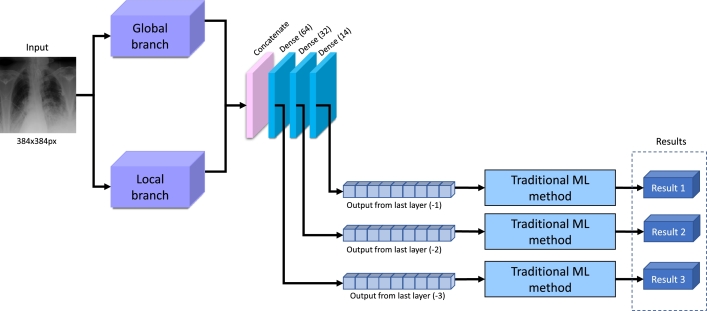
Overview of the experiment with fine-tuning and application of ML methods.

This part of the experiment is supposed to determine whether the CS treatment is needed, taking into consideration only a CXR image of the patient. The feature extractor is the main part, which is a pre-trained neural network from the previous step (see subsection 3.2.1). Instead of adding new layers to a pre-trained network, which would perform the binary classification, we applied traditional ML methods to the latent space of the three last layers of the neural network. The combination of NN and traditional ML has been successfully applied in some other works, for example, in [54], [34], [55]. This method was selected because fine-tuning pre-trained NN and adding several new layers to perform the classification can be ineffective since the dataset is relatively small. The traditional ML algorithms in our experiment were independently applied to the last three layers. Consequently, three different results were achieved. The scheme of the experiment is depicted in Fig. 7.

For the experiment, the following traditional ML algorithms [56] were evaluated: Random Forest (RF), Multilayer Perceptron (MLP), Logistic Regression (LR), Decision Tree (DT), *k*-Nearest Neighbors (kNN), Support Vector Machine (SVM), and AdaBoost.

An optimization technique, random search with cross-validation with five folds, is used to find the best hyper-parameter combination. The found optimal hyper-parameters for different ML methods and different outputs from the last layers are shown in Table 5.

**Table 5:** Detected hyper-parameters of machine learning methods for experiments with only CXR.

	Last layer (-1)	Last layer (-2)	Last layer (-3)
Random Forest	number of estimators: 1, max features: 12, max depth: 18, criterion: gini	number of estimators: 13, max features: 17, max depth: 7, criterion: gini	number of estimators: 3, max features: 7, max depth: 18, criterion: gini
MLP	solver: lbfgs, max iterations: 140, hidden layer size: 186	solver: sgd, max iterations: 38, hidden layer sizes: 172	solver: lbfgs, max iterations: 18, hidden layer sizes: 112
Logistic Regression	solver: saga, max iterations: 4, C: 3.5354	solver: lbfgs, max iterations: 16, C: 4.9495	solver: newton-cg, max iterations: 2, C: 9.7979
Decision Tree	max features: log2, max depth: 7, criterion: gini	max features: auto, max depth: 5, criterion: gini	max features: auto, max depth: 3, criterion: entropy
*k*-Nearest Neighbors	weights: uniform, number of neighbors: 14, algorithm: ball tree	weights: uniform, number of neighbors: 5, algorithm: auto	weights: uniform, number of neighbors: 5, algorithm: auto
SVM	kernel: poly, C: 0.5051	kernel: rbf, C: 2.0203	kernel: rbf, C: 1.8183
AdaBoost	number of estimators: 5, learning rate: 0.778	number of estimators: 24, learning rate: 0.445	number of estimators: 21, learning rate: 0.556

#### 3.3.2 Classification with mixed data

**Fig. 8:**
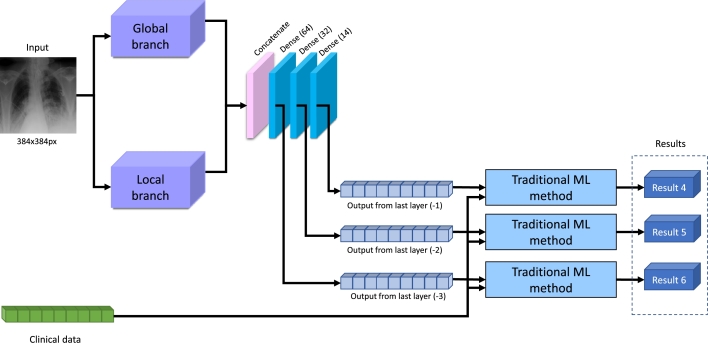
Overview of the experiment with a combination of CXR and clinical data.

The second part of the experiment aims to determine the necessity of CS, considering the combination of CXR images and clinical data. The experiment scheme is depicted in Fig. 8.

As can be seen, the CXR image is fed into our neural network, which is pre-trained on general pulmonary diseases, where the features are extracted. The outputs from the last layers are concatenated with the preselected clinical features (see section 3.1.2). Here, it is considered that additional information can improve the prediction accuracy of the necessity of CS treatment. And, as was described in the previous part, the traditional ML methods are applied independently to each pair layer output + clinical data. There were three experiments because the three last layers were used for the experiment.

As was presented in the previous part, the set of ML methods is used the same. Here also the optimization technique, such as random search with cross-validation (*n* = 5). The optimal hyper-parameters are found and introduced in Table 6.

**Table 6:** Detected hyper-parameters of machine learning methods for experiments with CXR images and clinical data.

	Last layer (-1)	Last layer (-2)	Last layer (-3)
Random Forest	number of estimators: 11, max features: 17, max depth: 14, criterion: entropy	number of estimators: 14, max features: 29, max depth: 14, criterion: gini	number of estimators: 5, max features: 21, max depth: 6, criterion: gini
MLP	solver: lbfgs, max iterations: 31, hidden layer size: 170	solver: adam, max iterations: 99, hidden layer sizes: 35	solver: adam, max iterations: 130, hidden layer sizes: 123
Logistic Regression	solver: lbfgs, max iterations: 3, C: 1.9599	solver: lbfgs, max iterations: 58, C: 9.7487	solver: lbfgs, max iterations: 54, C: 1.0051
Decision Tree	max features: auto, max depth: 4, criterion: entropy	max features: auto, max depth: 12, criterion: entropy	max features: log2, max depth: 13, criterion: entropy
*k*-Nearest Neighbors	weights: distance, number of neighbors: 14, algorithm: ball tree	weights: distance, number of neighbors: 14, algorithm: auto	weights: distance, number of neighbors: 14, algorithm: ball tree
SVM	kernel: rbf, C: 6.3819	kernel: rbf, C: 6.3819	kernel: rbf, C: 6.3819
AdaBoost	number of estimators: 21, learning rate: 0.5793	number of estimators: 23, learning rate: 0.3165	number of estimators: 15, learning rate: 0.8948

## 4 Results and discussion

This section represents the metrics used for comparing trained models, discusses the achieved results, and the future directions for further research.

### 4.1 Metrics

The metrics used are accuracy, precision, recall, balanced accuracy, and F1-score, which are usually used in evaluating classification tasks. The definitions of the mentioned metrics are shown below [35]:

(3) $$Accuracy=\frac{TN+TP}{TP+TN+FP+FN},$$

(4) $$Precision=\frac{TP}{TP+FP},$$

(5) $$Recall=Sensitivity=\frac{TP}{TP+FN},$$

(6) $$Specificity=\frac{TN}{TN+FP},$$

(7) $$F1=2\cdot \frac{Precision\cdot Recall}{Precision+Recall},$$

(8) $$Acc_{\mathrm{BAL}}=\frac{Specificity+Sensitivity}{2},$$

where *TN* – number of True Negative cases, *TP* – True Positives, *FP* – False Positives, *FN* – False Negatives.

### 4.2 Classification of chest X-ray data

This section introduces the results for predicting CS treatment necessity based on CXR images. Table 7 shows the results for different last layers output processed with some traditional ML methods. The best potential result for all metrics is 1, and the worst is 0. As can be seen, the most successful method is the RF, which was applied for the last layer (-3). The results are: accuracy is 0.7273, F1 is 0.7059, balanced accuracy is 0.7267, and ROC-AUC is 0.7247. However, MLP has the best result for recall 0.76. The kNN method from the last layer (-1) has the best precision result 0.73.

**Table 7:** Results for CS prediction using different last layers of NN for only CXR.

	Accuracy	F1	Precision	Recall	Balanced Acc	ROC-AUC
Last layer (-1) – Result 1						

Random Forest	0.7091	0.6923	0.6667	0.7200	0.7100	0.7100
MLP	0.6909	0.6531	0.6667	0.6400	0.6867	0.6760
Logistic Regression	0.5818	0.2581	0.6667	0.1600	0.5467	0.5627
Decision Tree	0.6364	0.6429	0.5806	0.7200	0.6433	0.6007
*k*-Nearest Neighbors	0.6727	0.5500	**0.7333**	0.4400	0.6533	0.6700
SVM	0.6000	0.5600	0.5600	0.5600	0.5967	0.5800
AdaBoost	0.5636	0.5000	0.5217	0.4800	0.5567	0.5547

Last layer (-2) – Result 2						

Random Forest	0.7091	0.6522	0.7143	0.6000	0.7000	0.6933
MLP	0.6364	0.6364	0.6364	0.6364	0.6364	0.6364
Logistic Regression	0.6727	0.6250	0.6522	0.6000	0.6667	0.6000
Decision Tree	0.5818	0.5818	0.5333	0.6400	0.5867	0.5647
*k*-Nearest Neighbors	0.6182	0.5882	0.5769	0.6000	0.6167	0.5907
SVM	0.6000	0.5217	0.5714	0.4800	0.5900	0.4380
AdaBoost	0.6545	0.5957	0.6364	0.5600	0.6467	0.6367

Last layer (-3) – Result 3						

Random Forest	**0.7273**	**0.7059**	0.6923	0.7200	**0.7267**	**0.7247**
MLP	0.6909	0.6909	0.6333	**0.7600**	0.6967	0.6693
Logistic Regression	0.6909	0.6383	0.6818	0.6000	0.6833	0.6533
Decision Tree	0.6000	0.6071	0.5484	0.6800	0.6067	0.6213
*k*-Nearest Neighbors	0.6545	0.6545	0.6000	0.7200	0.6600	0.6653
SVM	0.5818	0.4889	0.5500	0.4400	0.5700	0.4213
AdaBoost	0.5818	0.5490	0.5385	0.5600	0.5800	0.5633

For the other layers, the results are close to the ones mentioned above. For the last layer (-1), RF has the best results for accuracy (0.7091), F1 (0.6923), recall (0.72), balanced accuracy (0.71), and ROC-AUC (0.71). The RF performs better than other methods for the last layer (-2). It succeeded with an accuracy of 0.7091, F1 of 0.6522, precision of 0.7143, balanced accuracy of 0.70, and a ROC-AUC of 0.6933. DT method is the best for recall (0.64).

**Fig. 9:**
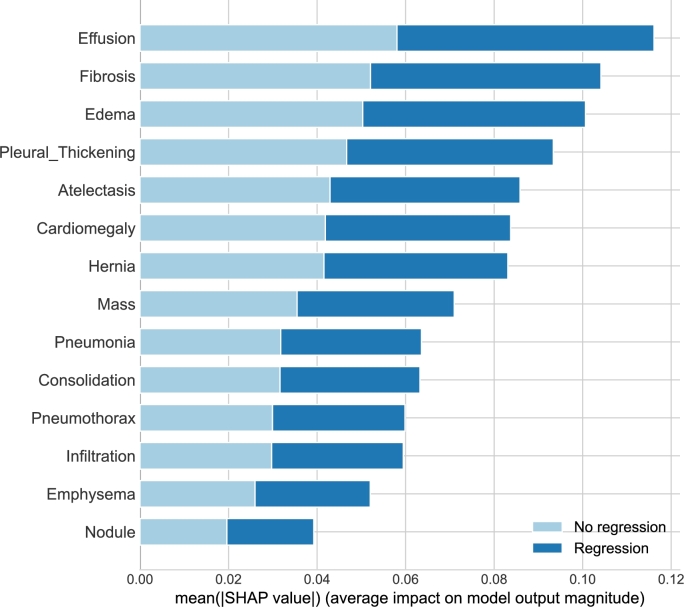
SHAP evaluation for the outputs of last layer (-1) of NN in experiments with only CXR.

Another interesting direction for investigation is the identification of the disease that has the most significant impact on the decision for CS treatment. As mentioned above, for this experiment, the used neural network was pre-trained on the large dataset ChestX-ray14, which is labeled for 14 different pulmonary diseases. The last layer output of this model is a vector of probabilities of various illnesses. This output is fed into selected traditional ML algorithms, and the decision on CS treatment is performed. To investigate, which values of NN output are the most significant for decision-making, the SHAP analysis was applied to explain the model. Fig. 9 shows the feature importance for the selected algorithm, in this case, for RF (accuracy is 0.7091). As can be seen, the highest values have the following diseases: Effusion, Fibrosis, and Edema. The other ones have less impact on output, but it is still high.

### 4.3 Classification of mixed data

Another conducted experiment has not only CXR image data but also additional clinical information about every patient (blood test, spirometry, and anamnesis). The results are shown in Table 8. Here, it is more difficult to determine the absolutely successful combination of one of the last layers and the ML method.

**Table 8:** Results for CS prediction using clinical data and different last layers of NN for CXR.

	Accuracy	F1	Precision	Recall	Balanced Acc	ROC-AUC
Last layer (-1) – Result 4						

Random Forest	0.7273	0.7059	0.6923	0.7200	0.7267	0.6840
Logistic Regression	0.6182	0.5714	0.5833	0.5600	0.6133	0.6547
Decision Tree	0.7636	**0.7869**	0.6667	**0.9600**	0.7800	0.7033
MLP	0.7455	0.7407	0.6897	0.8000	0.7500	0.7667
*k*-Nearest Neighbors	0.6727	0.6250	0.6522	0.6000	0.6667	0.6973
SVM	0.5818	0.4889	0.5500	0.4400	0.5700	0.7053
AdaBoost	0.6364	0.6296	0.5862	0.6800	0.6400	0.5967

Last layer (-2) – Result 5						

Random Forest	0.7455	0.7308	0.7037	0.7600	0.7467	0.7827
Logistic Regression	0.6182	0.5882	0.5769	0.6000	0.6167	0.6507
Decision Tree	0.6545	0.5957	0.6364	0.5600	0.6467	0.6467
MLP	**0.8000**	0.7442	0.8889	0.6400	**0.7867**	**0.8933**
*k*-Nearest Neighbors	0.6909	0.6383	0.6818	0.6000	0.6833	0.7093
SVM	0.5818	0.4889	0.5500	0.4400	0.5700	0.7053
AdaBoost	0.6364	0.5652	0.6190	0.5200	0.6267	0.5540

Last layer (-3) – Result 6						

Random Forest	0.7455	0.7308	0.7037	0.7600	0.7467	0.7727
Logistic Regression	0.6182	0.5882	0.5769	0.6000	0.6167	0.6413
Decision Tree	0.6727	0.6250	0.6522	0.6000	0.6667	0.6973
MLP	0.7636	0.6486	**1.0000**	0.4800	0.7400	0.8467
*k*-Nearest Neighbors	0.6909	0.6383	0.6818	0.6000	0.6833	0.7053
SVM	0.5818	0.4889	0.5500	0.4400	0.5700	0.7053
AdaBoost	0.6545	0.6122	0.6250	0.6000	0.6500	0.6047

However, the MLP for the last layer (-2) has the best results for accuracy (0.80), balanced accuracy (0.7867) and ROC-AUC (0.8933). On the other hand, DT for the last layer (-1) has achieved the best value of F1 (0.7869) and recall (0.96). Finally, MLP for the last layer (-3) has the absolute result for precision (1.0). Despite such different results, comparing Table 7, Table 8, it can be concluded that the combination of CXR and clinical data gives more accurate results than those performed only for CXR images.

Here, it can also be noticed that using outputs from the last layer can benefit the practical side. As less abstraction is used for decisions, it is easier to interpret the results. As mentioned in the previous section, the last layer of NN indicates the probability of a pulmonary disease.

Another point that would be considered is the importance of metrics. In some cases, paying attention to the rate of false negatives would be more essential since the missed therapy can be crucial for a patient. On the other side, considering several metrics can be more informative since they can give complete information regarding tested methods. Consequently, some weak sides can be identified. However, all these points should be consulted with experts (doctors) who will use the methodology.

Compared to our previous work [35], this approach has improved the capability of CS treatment prediction for more than 6% accuracy. Here, it can be concluded that additional information, such as CXR is useful in CS treatment prediction. Notably, the proposed methodology is more complex than the previous one, which indicates that this research field requires advanced processing methods. On the other hand, the problem of interpretation is arising. In previous work, the decision tree can be quickly drawn and the doctors can evaluate the correctness of the algorithm. However, in case of neural networks, it is getting more complicated, and several additional techniques are required to reveal the reasons for algorithm decisions.

### 4.4 Summary and future work

According to relatively good results, it can be concluded that the application of AI for preventive CS treatment can accurately recommend CS treatment to patients who can potentially benefit from the treatment and not recommend it to patients who, with a high probability, will not benefit from it and in this way, overtreatment will be avoided. We also conclude that combining the raw CXR image data with clinical data is significantly more accurate than just CXR images. Despite the application of relatively computation-demanding architectures, such as InceptionNet and Vision Transformer, the proposed architecture can be applied to real-world environments. This is proved by deploying the methodology into the internal hospital system. This work attempted to use the optimal input image size so that the details would be preserved, and it is not computationally demanding. On the other hand, if this methodology is applied in another hospital environment, it is necessary to consider the allowed latency, processing time, and frequency of usage. Depending on these points, the hardware should be chosen wisely. However, this work has some issues and requires some improvements.

Firstly, the relatively small custom dataset is used, which is why the overfitting problem can appear. As future work, this dataset is supposed to be extended, especially with the appearance of new mutations. Here it is also worth mentioning, that some patients were diagnosed with other methods, than anti-SARS-CoV-2 IgG testing, but with clinical symptoms, radiology image, and presence of anti-SARS-CoV-2 IgM antibodies. It indicates, that other clinical information should also be considered during patient examination and treatment prescription.

Secondly, in this work, the model was pre-trained on general pulmonary diseases. However, in future work, the model should be pre-trained also on COVID-19 datasets. It would make the NN more adapted to the problems related to COVID-19.

Thirdly, this paper utilizes the two methods of feature selection. However, the following work can extend this part too: more detailed analysis of clinical data using statistical methods and different parameter selection techniques is required. One of the possible directions is to use feature selection based on genetic algorithms as was proposed in [57].

Another raised problem is the interpretation of algorithm decision-making. Since the neural network is a kind of “black-box” it is necessary to apply the advanced interpretation techniques of such methods. This point will be addressed in future work.

Considering the complexity of ML/DL methods, it is reasonable to experiment with other techniques, for example, fuzzy similarities [58], [59]. Lastly, based on the received information on the patient and his treatment, the work can be extended by evaluating the severity and duration of the post-acute phase.

## 5 Conclusion

Prevention, diagnostics, and treatment of COVID-19 have improved significantly in recent years. However, severe cases may lead to long-term complications that may increase worldwide morbidity. One of the most severe complications is PF, which can be preventively treated using CS. Unfortunately, CS treatment has severe side effects, and the non-selected application of CS is considered harmful. Currently, no methodology can identify people who will benefit from the treatment and who will not.

This work introduced a novel, personalized AI-based methodology for CS treatment prescription during the post-acute phase of COVID-19. For the design of the methodology, we collected data from a total of 273 patients, which consist of more than 100 different parameters for each person. It includes data that were collected at the first examination of post-COVID treatment (blood tests, spirometry test, anamnesis, and CXR images), which is, regarding its detail, the largest reported dataset.

The proposed methodology is based on a neural network architecture pre-trained on a large dataset of pulmonary diseases ChestX-ray14. In this way, the neural network can extract the relevant features from input images. The first experiment is based on the processing of a CXR image by NN and the traditional ML algorithm with optimization, which was applied to get the final decision on the necessity of CS treatment. The second experiment has the additional part: clinical data concatenated to the NN output for the final decision.

Here results from CXR and a combination of CXR with clinical information were evaluated. We demonstrated that combining CXR and clinical information achieves significantly better results (78.7%).

The proposed methodology can be used in real-world practice. It can assist physicians in identifying individuals who need CS treatment. For sure, this field of research is promising. It can help improve the speed of the patient's examination, and more people would get personalized medical care and avoid overtreatment of patients who will not benefit from the treatment. The same principle may be applied to other diseases with a risk of PF development (e.g., organizing pneumonia, acute respiratory distress syndrome, or drug-induced lung involvement). This way, the pandemic of COVID-19 may give us lessons for future applications of AI.

## Informed consent statement

Patient consent was waived because the analysis was conducted on anonymized retrospective medical records.

## Institutional review board statement

The study was conducted in accordance with the Declaration of Helsinki, and approved by the Ethics Committee of University Hospital Olomouc and Faculty of Medicine and Dentistry (protocol code 228/21, approved 13.12.2021).

## CRediT authorship contribution statement

**Anzhelika Mezina:** Conceptualization, Investigation, Methodology, Writing – original draft. **Samuel Genzor:** Data curation, Formal analysis, Investigation, Writing – review & editing, Funding acquisition. **Radim Burget:** Funding acquisition, Project administration, Supervision, Writing – review & editing. **Vojtech Myska:** Data curation, Formal analysis, Writing – review & editing. **Jan Mizera:** Data curation, Formal analysis, Investigation, Writing – review & editing. **Aleksandr Ometov:** Project administration, Supervision, Writing – review & editing.

## Declaration of Competing Interest

The authors declare that they have no known competing financial interests or personal relationships that could have appeared to influence the work reported in this paper.
